# Herbal remedies and functional foods used by cancer patients attending specialty oncology clinics in Trinidad

**DOI:** 10.1186/s12906-016-1380-x

**Published:** 2016-10-21

**Authors:** Yuri N. Clement, Varune Mahase, Annelise Jagroop, Kelly Kissoon, Aarti Maharaj, Prashant Mathura, Chrys Mc Quan, Divya Ramadhin, Cherrista Mohammed

**Affiliations:** Pharmacology Unit, Faculty of Medical Sciences, University of the West Indies, St. Augustine, Trinidad and Tobago

**Keywords:** Prostate cancer, Breast cancer, Prostate cancer, Herbal remedies, Functional foods, *Annona muricata* L., *Beta vulgaris* L., *Daucus carota* L., *Triticum aestium* L., *Carica papaya* L.

## Abstract

**Background:**

Cancer is a major disease worldwide, and many patients use complementary and alternative treatments. The purpose of this study was to identify the herbal remedies and functional foods used as complementary medicine by prostate, breast and colorectal cancer patients at speciality care facilities in Trinidad. We also sought to determine how patients rated the efficacy of these modalities compared with conventional treatment.

**Methods:**

A descriptive, cross-sectional survey was conducted using an interviewer-administered pilot-tested *de novo* questionnaire during the period June to August 2012 at two speciality treatment centres on the island. Data was analysed using χ^2^ analyses.

**Results:**

Among the 150 patients who reported use of herbal remedies/functional foods, soursop (*Annona muricata* L.) was the most popular; with 80.7 % using the leaves, bark, fruit and seeds on a regular basis. Other common herbal remedies/functional foods included wheatgrass (*Triticum aestivum* L.), saffron (*Crocus sativus* L.) and *Aloe vera* (L.) Burm. f. The most commonly used functional foods were beetroot (*Beta vulgaris* L.), carrots (*Daucus carata* L.) and papaya (*Carica papaya* L.) used by 43.3 % of patients; and these were mostly blended as a mixture. Herbal remedies and functional foods were used on a daily basis and patients believed that this modality was equally (32.0 %) or more efficacious (14.7 %) than conventional treatment.

**Conclusions:**

This survey identified the most common herbal remedies and functional foods used among prostate, breast and colorectal cancer patients in Trinidad. Although functional foods rarely pose a problem, herbs may interact with conventional chemotherapy and physicians need to inform patients regarding probable herb-drug interactions.

**Electronic supplementary material:**

The online version of this article (doi:10.1186/s12906-016-1380-x) contains supplementary material, which is available to authorized users.

## Background

In Trinidad and Tobago the incidence of cancer and mortality rates are amongst the highest in the developing world [1]. The major ethnic groups on the islands are African and Asian Indian descendants with disproportionately higher incidences of prostate and breast cancers among African-Trinidadians [2], with poorer prognosis compared with their Asian Indian counterparts [3, 4]. It has also been shown that African-Caribbean men with prostate cancer living in the Caribbean had a 3.7-fold increased risk of death compared with their African-Caribbean born counterpart living the United States [5]. Similarly, African-Caribbean women with breast cancer had poorer prognosis than their African-American counterparts [6]. It has been suggested that poor survival rates may be due to diagnosis at advanced stages and failure to use multimodality as the first course of treatment [7, 8].

Although conventional interventions, including surgery, chemotherapy and radiation, are available, surveys elsewhere have shown that many cancer patients use various forms of complementary and alternative medicine [9, 10]. Previous surveys in Trinidad have also shown that there is high use of herbal remedies by patients accessing public health care facilities. In a survey at an asthma specialty care clinic in Trinidad we found that about 30 % of asthmatic patients used herbal remedies alongside their conventional medicines for symptomatic relief [11]. And, most of these patients reported beneficial effects. In another survey conducted at 22 primary healthcare clinics across the island we interviewed 759 patients being treated for hypertension and diabetes [12]. Likewise, a similar percentage of patients used herbal remedies together with their conventional medicines for self-management of their chronic disease(s) and for general health. Furthermore, in another survey at 16 public primary healthcare facilities, which included 265 patients who used herbal remedies, we focused on their perception of the efficacy of herbal remedies compared with conventional medicines [13]. Again, a significant proportion of users (86.8 %) reported that herbal remedies had either equal or greater efficacy compared with their conventional medicines.

A report from the National Cancer Registry of Trinidad and Tobago shows that prostate, breast and colorectal cancers have the highest incidence rates at 22 %, 14.6 % and 8.9 % respectively [14]. We therefore focused on these patients who indicated their use of herbal remedies and functional foods as complementary treatment. Our main objectives were to: i) determine the most common herbal remedies and functional foods used as complementary treatment in these patients, ii) assess patients’ perceived efficacy of herbal remedies and functional foods and iii) review the literature regarding the clinical and pharmacological evidence that could support the complementary use of identified herbal remedies and functional foods.

## Methods

The survey was descriptive and cross-sectional in design using a *de novo* pilot-tested questionnaire (Additional file 1) and adult patients were conveniently chosen at two specialized cancer treatment centres in Trinidad during the period June to August 2012. All participating patients signed their informed consent and the survey instrument was interviewer-administered, and we assumed that non-response was not a critical factor.

The survey instrument documented demographic details including gender, age, ethnicity, household income, education, area of residence; a brief medical history (type of cancer, grade of cancer, conventional treatments received), herbal and functional food use (frequency of use and perceived efficacy), patients’ subjective comparative ratings of efficacy of conventional treatment and herbal remedies/functional foods, physician’s awareness of patient use of herbal remedies/functional foods and the patient’s subjective attitude toward herbal remedy/function food use. Additionally, we recorded the mode of preparation of herbal remedies and functional foods.

The Statistical Package for the Social Sciences (SPSS) Program, Version 20.0 was used for data input and analysis. Chi-square analyses were performed to determine statistically significant associations between demographic characteristics and cancer type, and herbal remedies and functional foods use and cancer type; a p-value <0.05 was considered statistically significant.

## Results

One hundred and fifty patients agreed to participate; they were mostly female (102 or 68 %), with breast cancer (88 or 58.7 %), of African descent (78 or 52 %), over 40 years of age (137 or 91.3 %), with at least secondary level education (98 or 65.3 %) and earned under US$1000 monthly (81 or 54 %), Table 1. Although a significant number of patients (44 or 29.3 %) did not know the stage of cancer, most of the other patients (92 or 61.3 %) had progressed to grade 2 or above (Table 1). Most patients (103 or 68.7 %) received at least one form of conventional treatment: chemotherapy alone (45 or 30 %), surgery and chemotherapy (28 or 18.7 %) and chemotherapy, radiotherapy and surgery (30 or 20 %). Fifty-five herbal remedies, supplements and functional foods were identified in the survey. On average, patients used 2.7 medicinal herbs/functional foods; and most patients (103 or 68.7 %) consumed these at least once a day. Soursop (*Annona muricata* L.) was the most common herb (121 or 80.9 %) in all types of cancers, and all parts of the plant were used (Table 2). The leaves, bark and seeds were used to make infusions and decoctions, and the ripe fruit was eaten raw, juiced or blended. Two patients consumed either boiled or steeped soursop seeds. Other commonly used herbs included wheatgrass *(Triticum aestivum* L.), saffron (*Crocus sativus* L.), *Aloe vera* (L.) Burm f., garlic (*Allium sativum* L.) and ginger (*Zingiber officinale* Roscoe). A significant number of patients used blended or pureed mixed vegetable juices, which included beetroot (*Beta vulgaris* L.), carrots (*Daucus carota* L.), cucumber (*Cucumis sativus* L.) and papaya (*Carica papaya* L.). The highest levels of herbal remedy and functional foods use was observed amongst patients with breast cancer; whereas prostate cancer patients had the lowest levels of use with no reported use of either carrot (*Daucus carota* L.) or saffron (*Crocus sativus* L.). Breast cancer patients were more likely to use beetroot (*p =* 0.04) and carrot (*p =* 0.006), whereas prostate cancer patients were more likely to use noni (*p =* 0.014), Table 2.

**Table 1:** Demographic details for patients with breast, prostate and colorectal cancer

Demographic variable	Cancer type			Total *N =* 150 (%)	Pearson’s Chi-square *p* value
	Breast	Prostate	Colorectal		
Sex					
Female	88	0	14	102 (68.0)	<0.001*
Male	0	36	11	47 (31.3)	
Age					
18–29	1	0	0	1 (0.7)	0.020*
30–39	12	0	0	12 (8.0)	
40–49	14	3	8	25 (16.7)	
50–59	31	8	3	42 (28.0)	
60–69	18	17	12	47 (31.3)	
> 70	12	8	3	23 (15.3)	
Ethnicity					
African	44	20	14	78 (52.0)	
Indian	27	9	8	44 (29.3)	
Mixed	16	6	4	26 (17.3)	0.814
Other	1	1	0	2 (1.3)	
Highest education					
None	1	0	1	2 (1.3)	0.632
At least Primary school	25	10	8	43 (28.7)	
At least Secondary school	34	17	9	60 (40.0)	
University	24	7	5	36 (24.0)	
Postgraduate	1	1	0	2 (1.3)	
Other	3	2	3	8 (5.3)	
Stage					
Do not know	24	12	9	45 (30.0)	0.079
1	8	1	3	12 (8.0)	
2	25	11	1	37 (24.7)	
3	18	4	3	25 (16.7)	
4	13	7	10	30 (20.0)	
Remission	1	0	0	1 (0.7)	
Monthly income (US$)					
< 1000	43	24	14	71 (54.0)	0.210
1001–2000	10	5	4	19 (12.7)	
> 2000	0	1	0	1 (0.7)	
Non response	35	6	8	49 (32.7)	

*p < 0.05

**Table 2:** Common herbal remedies and functional foods used by cancer patients

Herb/functional food	Cancer type			Total *N =* 150 (%)	Pearson’s Chi-square *p* value
	Breast (*n =* 88)	Prostate (*n =* 36)	Colorectal (*n =* 25)		
Soursop (*Annona muricata* L.) Family: Annonaceae	70	29	22	121 (80.7)	0.878
Beet root (*Beta vulgaris* L.) Family: Amaranthaceae	23	3	3	29 (19.3)	0.040*
Carrot (*Daucus carota* L.) Family: Apiaceae	18	0	2	20 (13.3)	0.006*
Wheatgrass (*Triticum aestivum* L.) Family: Poaceae	12	1	4	17 (11.3)	0.173
Papaya (*Carica papaya* L.) Family: Caricaceae	12	4	0	16 (10.7)	0.191
Saffron (*Crocus sativus* L.) Family: Iridaceae	12	0	3	15 (10.0)	0.069
Aloes (*Aloe vera* (L.) Burm. f.) Family: Xanthorrhoeaceae	9	3	0	12 (8.0)	0.239
Cucumber (*Cucumis sativus* L.) Family: Cucurbitaceae	10	0	1	11 (7.3)	0.067
Lemon (*Citrus x limon* (L.) Osbeck) Family: Rutaceae	7	1	2	10 (6.7)	0.562
Noni (*Morinda citrifolia* L.) Family: Rubiaceae	2	6	2	10 (6.7)	0.014*
Ginger (*Zingiber officinale* Roscoe) Family: Zingiberceae	7	0	3	10 (6.7)	0.150
Garlic (*Allium sativum* L.) Family: Amaryllidaceae	7	3	0	10 (6.7)	0.324

*p < 0.05

Most patients (131 or 87.4 %) used herbs and functional foods because they believed that these modalities were effective in destroying cancer cells. Most patients (123 or 82 %) reported side-effects to conventional treatments, with the most common being nausea and vomiting (63 or 42 %), pain (35 or 23.3 %), hair loss (34 or 22.7 %), skin and nail changes (27 or 18 %) and fatigue (14 or 9.3 %). However, only a small number of patients (39 or 26.0 %) used this modality to counteract the side effects of chemotherapy and radiotherapy. And, in 59 patients (39.3 %) their use of herbal remedies and functional foods was based on testimonials from other cancer patients. Most of the information was obtained from family members and friends (54 or 36.0 %) and the mass media, including the internet (25 or 16.7 %).

Although there were no objective clinical measurements to determine efficacy of these herbal remedies/functional foods, 71 patients (47.3 %) believed that supplementation was beneficial in the management of their condition, with about equal numbers (66 or 43.7 %) reporting uncertainty or no beneficial effects; and the other 13 patients (8.7 %) did not respond. Despite this, 101 patients (or 67.4 %) stated that they were either satisfied or highly satisfied with the use of herbs or functional food for both maintaining their health and for cancer treatment; and, most patients (135 or 90.0 %) indicated that they would continue using herbal remedies and functional foods in the long term. And this was supported by the significant number of patients (100 or 66.7 %) who believed that herbal remedies and functional foods were either equally or more efficacious than conventional treatment.

Just about half the number of patients in the survey (77 or 51.3 %) did not inform their attending physicians about their use of herbal remedies and functional foods; and, this behaviour was not influenced by demographics, but by the perception that physicians would discourage them. Most patients believed that their understanding of the scientific evidence and positive testimonials from other cancer patients were sufficient to support their use of this form of complementary medicine.

## Discussion

To our knowledge this is the first observational study in Trinidad to determine the complementary use of medicinal herbs and functional foods among patients diagnosed with breast, prostate and colorectal cancer. The study at two clinical sites on the island found that although most patients reported being at Stage II or above and treated with chemotherapy, surgery and/or radiotherapy, a significant number of patients were not aware of their stage of cancer.

We found a wide range of herbal remedies and functional foods being used by respondents for cancer treatment, health maintenance and to counteract side effects of conventional treatment. As in other studies [10, 15], most patients in our survey believed that herbs would destroy cancer cells, with fewer side effects than conventional therapy. Most patients used herbal medicines and functional foods on a daily basis in the diet alongside conventional therapies. The herbal treatments were usually consumed as a juice or a tea (infusion) using the leaves. Some patients use the herbs concomitantly with conventional therapy and some take it before and after the course of conventional treatment.

Patients were generally satisfied with the efficacy of herbs/foods used and believed that these therapies were equally or more effective than conventional medicine. Although most patients indicated that they would continue to use or recommend the use of herbs/foods, less than half stated that they benefited from the use of these herbs/foods at that point in time.

A review of the literature revealed limited clinical evidence to support the use of most of the herbal remedies and functional foods cited in this survey. However, we assessed the literature with regards to *in vitro* and *ex vivo* research to determine whether the herbal remedies and functional foods demonstrate anticancer properties that may lend support to their use in our patient sample.

Soursop (*Annona muricata* L.) was the most popular medicinal plant among respondents; with the leaves, bark, fruit and seeds being used. The seeds, leaves and bark were boiled to made decoctions, and the ripe fruit was eaten fresh or juiced. In a recent nationwide survey in Trinidad we also found that the leaves of soursop were used traditionally as “cooling/cleanser” and for the treatment of hypertension [16]. Several studies have identified cytotoxic acetogenins in the leaves and fruit of *Annona muricata* L [17–19]. Various extracts of *Annona muricata* L. have shown anti-proliferative activity *in vitro* by cell cycle arrest and apoptosis in prostate, colon and breast cancer cell lines [20–22]. However, to date there is no evidence from placebo-controlled clinical trials to support its use in the treatment of any type of cancer.

Beetroot (*Beta vulgaris* L.), juiced or blended, was the second most popular functional food used among this group of cancer patients. An extract of beetroot was shown to be cytotoxic in androgen-independent human prostate cancer cells and estrogen receptor-positive human breast cancer cells [23]. Additionally, a synergistic anti-proliferative effect was observed in breast and prostate cancer cell lines when treated with a combination of red beetroot extract and doxorubicin [24]. It has been suggested that betanine and betalain, major pigment constituents of beetroot, may be responsible for the cytotoxic activity. Again, there are no published clinical studies to establish a role for beetroot in the treatment of cancer.

Like beetroot, a common trend is the use of carrot juice for the prevention and treatment of a variety of cancer types. It has been suggested that bioactive compounds, such as β-carotene and polyacetylenes, are responsible for its cancer-protective properties. One of the first observational studies by Longnecker and colleagues [25] established an association between carrot consumption and a reduced risk of breast cancer. This study included 3543 cases and 9406 controls and it showed that consumption of carrots more than twice weekly reduced the odds of developing cancer by almost half. A recent meta-analysis of observational studies also showed that carrot intake was inversely related to the incidence of prostate cancer [26].

Oxidative stress is known to induce cancer and a recent study in overweight breast cancer survivors showed that the daily consumption of carrot juice increased plasma carotenoid levels, this would subsequently reduce oxidative stress and it has been postulated that this would reduce the risk of recurrence of cancer [27]. Studies in human colon and breast cancer cell lines demonstrated anti-proliferative effects of extract of carrot oil by modulating various mediators of apoptosis and cell cycle arrest [28, 29].

Wheatgrass (*Triticum aestivum* L.) was another popular remedy used mostly by breast and colorectal cancer patients in our study. Although there were no clinical studies to support its use, there are a few *in vitro* studies using breast and colorectal cancer cell lines. Wheatgrass is fermented by gut microflora to secondary products, and it has been proposed that these compounds possess significant antineoplastic activity. Non-fermented and fermented wheatgrass extract significantly decreased growth in a colon cancer cell line [30]. These results were corroborated in another laboratory study which showed that fermented wheatgrass extract inhibited growth and increased apoptosis in two different colon cancer cell lines [31]. More recently, it was shown that the combination of cisplatin and wheatgrass extract had a synergistic effect to inhibit growth in a breast cancer cell line and modulate apoptosis and proliferation-associated genes [32].

The use of papaya (*Carica papaya* L.) was also common among our patients with leaves being used to make a tea and the fruit being juiced or blended. The aqueous extract of fresh papaya leaves was tested against various cancer cell lines and human peripheral blood mononuclear cells to determine whether it exhibited cytotoxic activity and inhibitory effects on various biomarkers of inflammation and apoptosis [33]. The results showed that papaya extract significantly inhibited growth of cancer cells and down-regulated the expression of pro-inflammatory cytokines IL-2 and IL-4. Although the use of papaya in cancer treatment is commonly practiced, a review of the literature did not unearth any observational or interventional clinical studies which supported the use of papaya in cancer treatment.

Although the Indian spice saffron (*Crocus sativus* L.) was also popular among our patients, we suspect that the spice used may in fact be mixed with other spices such as turmeric, which is a known cultural culinary/traditional medicine practice among East Indians in Trinidad. Crocin, the major constituent of saffron, was shown to have a dose-dependent anti-proliferative effect against three colorectal cancer cell lines and a non-small cell lung cancer cell line whilst not having any effect on normal cells [34]. In another *in vitro* experiment crocetin, the main metabolite of crocin, was anti-proliferative and prevented the migration of a highly invasive breast cancer cell line by down-regulating the expression of matrix metalloproteinases [35]. Extracts have also been shown to have anti-proliferative effects against aggressive prostate cancer cell lines by mechanisms which include down-regulation of metalloproteinases, DNA fragmentation and induction of mediators of apoptosis [36, 37].

This study was limited due to its cross-sectional nature and the short time frame and we were not able to follow-up patients to determine the impact of supplementation on clinical outcomes. Our access was limited to patients attending public healthcare facilities who generally were at the lower economic strata with less disposable income to make “out-of-pocket” purchases for commercial products. We also limited our target population to breast, prostate and colorectal cancer patients and there may be differences in the pattern of herbal remedy and functional food use in patients with other types of cancers. However, despite these limitations the study highlighted the most common herbal remedies and functional foods used in the most common cancers in patients accessing public healthcare in Trinidad.

## Conclusions

This study unearthed the herbal remedies and functional foods used by patients with the most prevalent cancers in Trinidad. It was not surprising that soursop (*Annona muricata* L.) was the most common herbal remedy, as the herb has strong traditional use on the island. We also found high use of juices made from vegetables, which were used on a daily basis by most patients. As in our previous surveys in Trinidad [11–13] women used more of herbal remedies/functional foods than men, especially beetroot and carrots. Most of the scientific evidence of antineoplastic activity of these plants and functional foods comes from *in vivo* and *ex vivo* studies. However, despite the lack of clinical evidence most patients felt or believed that these remedies are equally or more efficacious than conventional treatments.

## Additional files

Additional file 1: Survey questionnaire. (DOC 45 kb)
Additional file 2: SPSS data file. (SAV 83 kb)
Additional file 3: Ethics Approval letter. (JPG 2429 kb)
