# Supplementary material for: Herbal remedies and functional foods used by cancer patients attending specialty oncology clinics in Trinidad
Source: BMC Complement Altern Med. 2016 Oct 21;16:399. doi: 10.1186/s12906-016-1380-x (PMC5073821; doi:10.1186/s12906-016-1380-x)
Supplement: Additional file 1: — Survey questionnaire. (DOC 45 kb) [file 12906_2016_1380_MOESM1_ESM.doc]

**PUBLIC HEATH & PRIMARY CARE Student Research Project 2012**

**Faculty of Medical Sciences**

**University of the West Indies**

**St. Augustine**

***INVESTIGATION OF HERBAL MEDICINE USE AND PERCEIVED EFFICACY AMONG ADULT PROSTATE, BREAST AND COLORECTAL CANCER OUTPATIENTS, ATTENDING SPECIALTY CARE FACILITIES IN TRINIDAD***

Protocol Number ______ Site: _____________________

**DEMOGRAPHIC INFORMATION**

1. Sex: Male ⁪ Female ⁪ 2. Age: _______
2. Marital Status: ______________________
3. Highest Level of Education Achieved: ________________________
4. Present employment position: _______________________________
5. Area of Residence: ________________________________________
6. Monthly Income: < $3000/month ⁪ $3001 - $6000/month ⁪

$6001- $9000/month ⁪ $9001 - $12000/month ⁪ > $12000/month ⁪

8. Ethnicity: African ⁪ Indian ⁪ Mixed ⁪ Other ⁪

1. Religion: Christian ⁪ Muslim ⁪ Hindu ⁪ Other ⁪

**TYPE OF CANCER AND THE CONVENTIONAL MEDICINE USED**

1. Type of Cancer Diagnosed :

Prostate ⁪ Breast ⁪ Colorectal ⁪

1. At what stage of Cancer are you? _______________________
2. How long have you been diagnosed with cancer? ____________
3. How long have you begun receiving treatment for this cancer?

________________________________________________________

1. What **type(s)** of treatment have you been received for cancer?

Surgery ⁪

Drug therapy ⁪

Radiotherapy ⁪

Other ⁪ _________________________

1. How long have you been undergoing this current type of treatment? _______________________________________________________
2. Have you experienced any side effects while undergoing this cancer treatment? If yes, please list.

   ________________________________________________________________________________________________________________________________________________

**HERBAL MEDICINE INFORMATION**

1. Have you ever taken any form of complementary or alternative medicines to treat your cancer, besides those prescribed by your medical doctor? Yes ⁪ No ⁪

**if no, end the interview here**

1. If yes, please list the types of herbal remedies, energy therapies (electromagnetic), physical therapies or even spiritual therapies, etc. that you currently use or have used in the past.

________________________________________________________________________________________________________________________________________________________________________________________________________________________________________________________________________________________

1. If you are using herbal remedies, how long have you been taking them? ___________________________________________________
2. Do you use herbal remedies for:

Cancer treatment ⁪

To combat side effects of conventional cancer treatment ⁪

1. How do you obtain these herbal/alternative medications? Supermarket/ Market ⁪ Relative/Friend ⁪ Home Garden ⁪

Herbalist ⁪ Pharmacy ⁪ Other ⁪ ___________

1. If you prepare your own remedies, please give a description of the method of preparation and how you administer it. ________________________________________________________________________________________________________________________________________________________________________________________________________________________
2. How did you find out about herbal remedies for the treatment of cancer and/or side effects of anticancer treatment?

- Healthcare personnel at hospital ⁪
- Friends ⁪
- Family members ⁪
- Alternative medicine practitioner ⁪
- Mass media (TV, newspaper, radio, magazines) ⁪
- Church/religious group ⁪
- Other patients ⁪
- Other ⁪ ___________________________

1. How often do you use herbal remedies to treat your cancer or combat anticancer treatment side effects? ________________________________________________________________________

25. If you use herbal remedies to treat conventional cancer treatment side-effects, list the specific herbs used to treat the specific cancer treatment.

________________________________________________________

________________________________________________________________________________________________________________________________________________________________________________________________________________________

1. Do you use herbal remedies:
   1. When you complete conventional treatment ⁪
   2. With conventional medicine ⁪
   3. And have stopped use of conventional medicine. ⁪
2. If you visit an herbal medicine practitioner, how often do you do so and what is the average cost per visit? ________________________________________________________________________
3. What were your reasons for using herbal medication rather than depending on conventional medication?

________________________________________________________________________________________________________________________________________________________________________________________________________________________

1. What benefits of herbal medication do you hope to derive from its use and have you achieved any benefit while using herbs?

________________________________________________________________________________________________________________________________________________

1. Have you experienced any side-effects to these herbal medicines? Yes ⁪ No ⁪

If yes, please list and indicate how they were treated. ________________________________________________________________________________________________________________________________________________________________________________________________________________________

1. How would you rate the herbal medicine you use to the prescribed conventional cancer treatments?

Much more effective ⁪ Somewhat effective ⁪

Similarly effective ⁪ Slightly less effective ⁪

Not effective at all ⁪

1. From your experience, what is your level of satisfaction with herbal remedies in the treatment of cancer/combating anticancer side effects?

Highly dissatisfied ⁪ Slightly dissatisfied ⁪

Cannot say ⁪ Slightly satisfied ⁪

Highly satisfied ⁪

1. Would you continue to use these herbal medicines for cancer treatment/combating anticancer treatment side-effects? Yes ⁪ No ⁪
2. Would you recommend herbal remedies to someone with your type of cancer? Yes ⁪ No ⁪
3. Did any of the herbal remedies cause you to stop taking conventional cancer treatment? Yes ⁪ No ⁪

If Yes, please explain: ________________________________________________________________________________________________________________________________________________________________________________________________________________________

1. Did you inform your medical doctor that you are using herbal medication as a primary form of cancer treatment? Yes ⁪ No ⁪

If no, please explain:

________________________________________________________________________________________________________________________________________________

*Thank you for your kind participation*
