# Supplementary figures and images for: Herbal remedies and functional foods used by cancer patients attending specialty oncology clinics in Trinidad
Source: BMC Complement Altern Med. 2016 Oct 21;16:399. doi: 10.1186/s12906-016-1380-x (PMC5073821; doi:10.1186/s12906-016-1380-x)

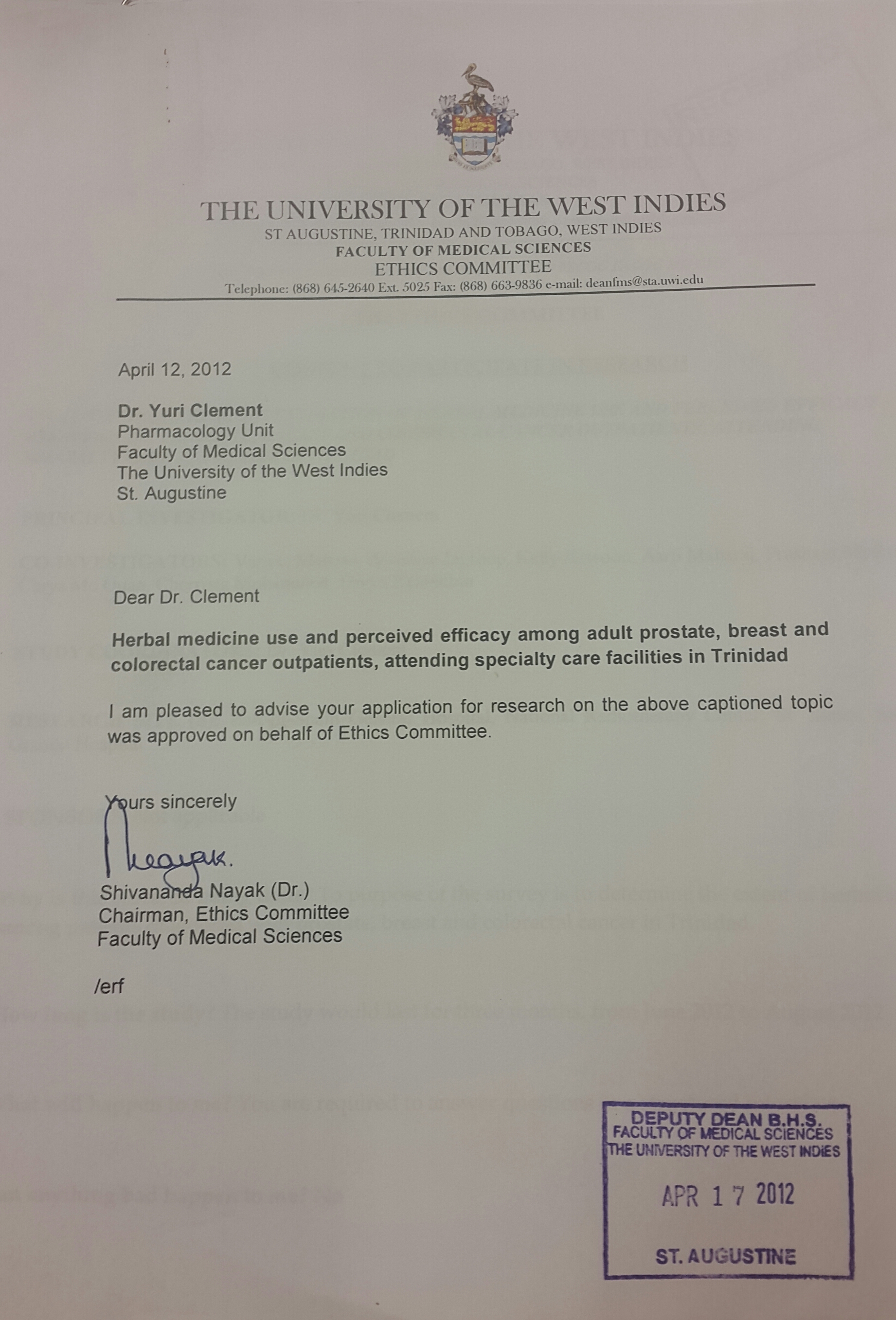

Supplement: Additional file 3: — Ethics Approval letter. (JPG 2429 kb) [file 12906_2016_1380_MOESM3_ESM.jpg]
